# Expression of KK-LC-1, a cancer/testis antigen, at non-tumour sites of the stomach carrying a tumour

**DOI:** 10.1038/s41598-018-24514-9

**Published:** 2018-04-17

**Authors:** Takashi Fukuyama, Nobue Futawatari, Rui Yamamura, Taiga Yamazaki, Yoshinobu Ichiki, Akira Ema, Hideki Ushiku, Yatsushi Nishi, Yoshihito Takahashi, Toshikazu Otsuka, Hitoshi Yamazaki, Wasaburo Koizumi, Kosei Yasumoto, Noritada Kobayashi

**Affiliations:** 1 Division of Biomedical Research, Kitasato University Medical Center, Kitamoto, Japan; 2 Department of Surgery, Sagamihara National Hospital, Sagamihara, Japan; 3 Second Department of Surgery, University of Occupational and Environmental Health, Kitakyushu, Japan; 4 Department of Surgery, School of Medicine, Kitasato University, Sagamihara, Japan; 5 Division of Surgery, Kitasato University Medical Center, Kitamoto, Japan; 6 Division of Gastroenterology, Kitasato University Medical Center, Kitamoto, Japan; 7 Division of Pathology, Kitasato University Medical Center, Kitamoto, Japan; 8 Department of Gastroenterology, School of Medicine, Kitasato University, Sagamihara, Japan; 9 Moji Hospital, Kitakyushu, Japan

## Abstract

Kita-Kyushu lung cancer antigen-1 (KK-LC-1) is a cancer/testis antigen (CTA) and predominant target for cancer immunotherapy. Our previous study indicated that KK-LC-1 was expressed in 82% of gastric cancers, and also in 79% of early stage of gastric cancers, with a correlation to *Helicobacter pylori (H. pylori)* infection. In addition, we found that KK-LC-1 was occasionally expressed at non-tumour sites of stomachs carrying tumours. Here, we investigated the characteristics of KK-LC-1 expression at non-tumour sites and the clinical utility of these phenomena. The gene expression of KK-LC-1 was detected at the non-tumour sites including pyloric glands. The most detectable corpus/gland subset had a KK-LC-1 expression rate of 77% in the pyloric gland of the lower corpus where *H. pylori* preferentially exists. KK-LC-1 expression rates were 67% or 32% with or without intestinal metaplasia, which also induced by *H. pylori*, respectively. Consequently, KK-LC-1 would be detected at the pre-cancerous condition of the stomach, and may be a useful marker to predict gastric cancer.

## Introduction

Gastric cancer is the third leading cause of cancer-related death worldwide after lung and liver cancers^1^. Particularly in Japan, gastric cancer has the second highest incidence rate of all cancers, and almost all cases are caused by *Helicobacter pylori (H. pylori)* infection^2^. Detection of *H. pylori* infection may reduce the risk of gastric cancer. Furthermore, ABC diagnosis employing an anti-*H. pylori* antibody and measuring the serum level of pepsinogen I/II is an approach for risk diagnosis of gastric cancer^3^. However, they are not accurate and indicate only 1–5% occurrence of gastric cancer in high risk groups. Therefore, more precise methods are needed for risk diagnosis^4^.

Several tumour-associated antigens have been identified in various human cancers^5^. These antigens are classified into four categories, excluding extrinsic viral antigens, as follows: cancer/testis antigens (CTAs), differentiation antigens, amplification or overexpression antigens, and tumour-specific mutated antigens recently defined as neo-antigens. CTAs are especially attractive targets for immunotherapy because they are not or minimally expressed in normal tissues except for the testis, but are aberrantly expressed in a range of human cancers^6^. Therefore, immune targeting of these antigens is thought to have negligible adverse side effects. Moreover, they may be advantageous molecules for systemic diagnosis of cancer because of their specific expression patterns. However, data on the expression rate of individual CTAs are not sufficient for diagnostic applications.

Kita-Kyushu lung cancer antigen-1 (KK-LC-1), also known as CT83 and Cxorf61, is a CTA that has epitope peptides recognised by cytotoxic T lymphocytes (CTLs). When CTLs against KK-LC-1 accumulate predominantly among tumour-infiltrating lymphocytes (TILs), adaptive immunotherapy using TILs leads to a good response^7^. KK-LC-1 maps to chromosome Xq22, it is not expressed in normal tissues except for the testis, but is expressed in 33% of non-small cell lung cancers^8,9^. Furthermore, KK-LC-1 is frequently expressed in 82% and 75% of gastric cancer and triple negative breast cancer (TNBC) patients^10,11^. According to these data, KK-LC-1 could be categorised as a CTA, but the expression rate of KK-LC-1 in gastric cancer and TNBC is not consistent with the traditional concept of CTAs except for melanoma antigen (MAGE)-A1 in hepatic cell carcinoma (HCC).

We previously reported that *H. pylori* infection induces expression of specific CTAs in addition to causing malignant transformation of host cells^12^. In clinical gastric tumours, KK-LC-1 expression correlates with *H. pylori* infection^13^. These results suggest that specific CTA expression may correlate with the initial cancer-causing event. Therefore, CTAs could be new candidates for immunotherapy to predict and diagnose gastric cancer.

KK-LC-1 is expressed during the early stage of gastric cancer, indicating that KK-LC-1 expression occurs at malignancy^14^. In this previous study, we found a certain expression rate of KK-LC-1, but no other CTAs, at non-tumour sites of a stomach carrying a tumour. In this study, we detected the expression of KK-LC-1 at non-tumour sites of stomachs carrying tumours and assessed the clinical utility of KK-LC-1 for surveillance of gastric cancer occurrence.

## Results

### CTA expression of specimens at tumour sites

Reverse transcription-polymerase chain reaction (RT-PCR) was used to determine the fractions of the tumour site and non-tumour sites, which expressed specific CTA genes. In gastric cancer tumours, 66 (80.5%) out of 82 patients had expression of KK-LC-1, which was markedly higher than 22 (26.8%), 34 (41.5%), 17 (20.7%), 18 (22.0%), and 14 (17.1%) patients with MAGE-1, MAGE-A3, MAGE-A4, Synovial sarcoma, X break point 4 (SSX4), and New York esophageal squamous cell carcinoma-1 (NY-ESO-1) expression (Table 1).

**Table 1:** Expression of CTAs in tumour area of 82 specimens.

CTA	Positive	Negative	%
KK-LC-1	66	16	80.5
MAGE-A1	22	60	26.8
MAGE-A3	34	48	41.5
MAGE-A4	17	65	20.7
SSX4	18	64	22.0
NY-ESO-1	14	68	17.1

### Specific expression of KK-LC-1 at non-tumour sites

**Figure 1:**
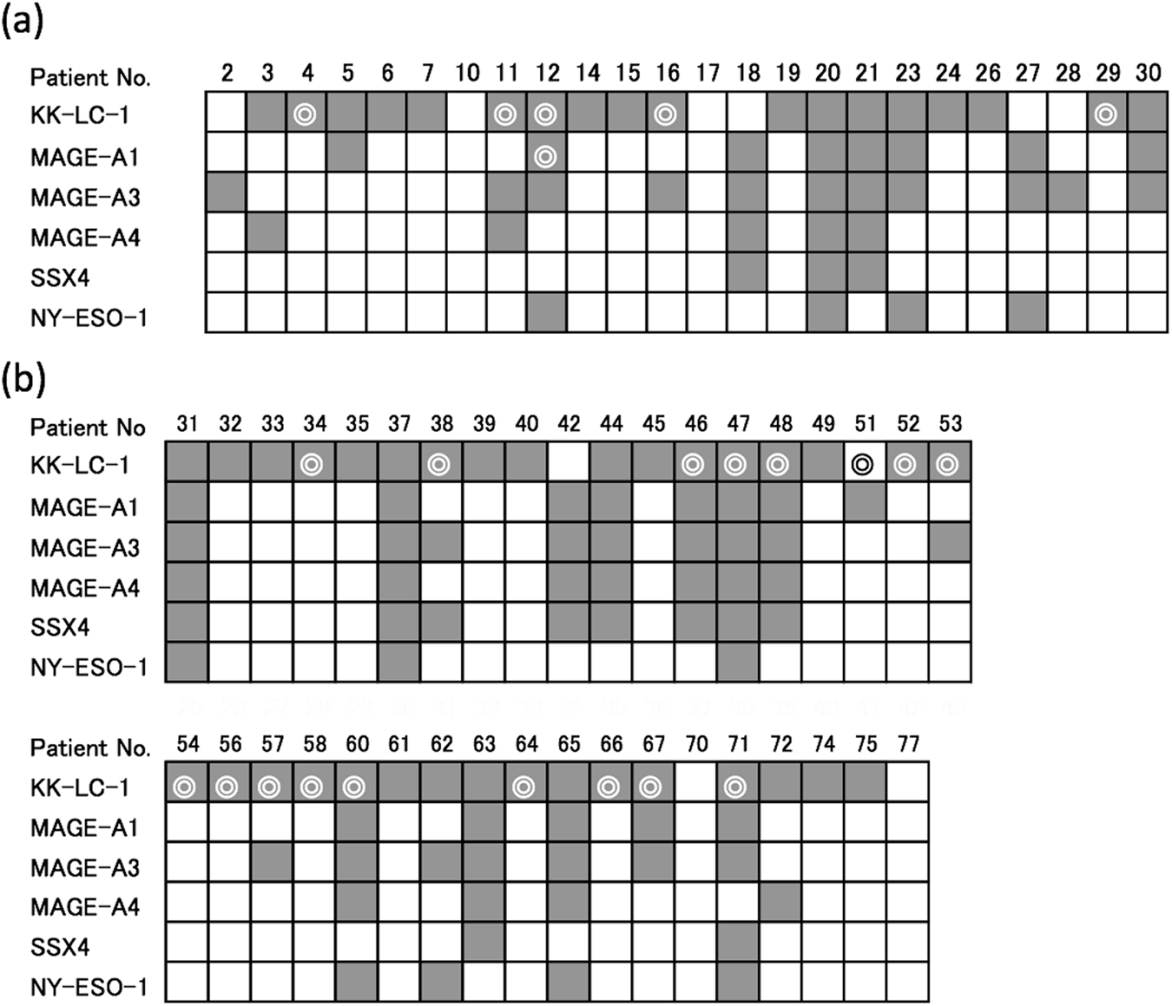
Expression of CTAs in tumour and normal areas of each gastric cancer patient. Each gastric specimen carrying a tumour was sampled in one tumour area and one (**a**) or two (**b**) non-tumour areas that were distant from the tumour and random. Each specimen was evaluated for the expression of CTAs in a tumour area and non-tumour area(s). Open or closed squares indicate no expression or expression of the CTA in a tumour area. Double circles represent expression of the CTA in a non-tumour area (**a**) or one or both non-tumour areas (**b**).

We examined 24 specimens in which CTA expression was detected at the tumour site and a non-tumour site (Fig. 1a). At tumour sites, 18 (75%), eight (33%), 11 (46%), five (21%), three (13%), and four (17%) patients had KK-LC-1, MAGE-A1, MAGE-A3, MAGE-A4, SSX4, and NY-ESO-1 expression. At non-tumour sites, expression of KK-LC-1 and MAGE-A1 was detected in five and one out of 24 patients, from whom the tumour site and one non-tumour site was sampled, respectively, of which tumour sites in the same patients all expressed each CTA (Fig. 1a). We examined another 37 specimens in which CTA expression was detected at the tumour site and two non-tumour sites (Fig. 1b). At tumour sites, 33 (89%), 13 (35%), 16 (43%), 11 (30%), 10 (27%), and seven (19%) specimens expressed KK-LC-1, MAGE-A1, MAGE-A3, MAGE-A4, SSX4, and NY-ESO-1, respectively. Seventeen specimens expressed KK-LC-1 at one or more non-tumour sites. Sixteen out of 17 specimens, in which KK-LC-1 was detected at non-tumour sites, expressed KK-LC-1 at tumour sites. None of the other CTAs were expressed at non-tumour sites.

### KK-LC-1 detection rates at non-tumour sites of gastric cancer specimens

**Figure 2:**
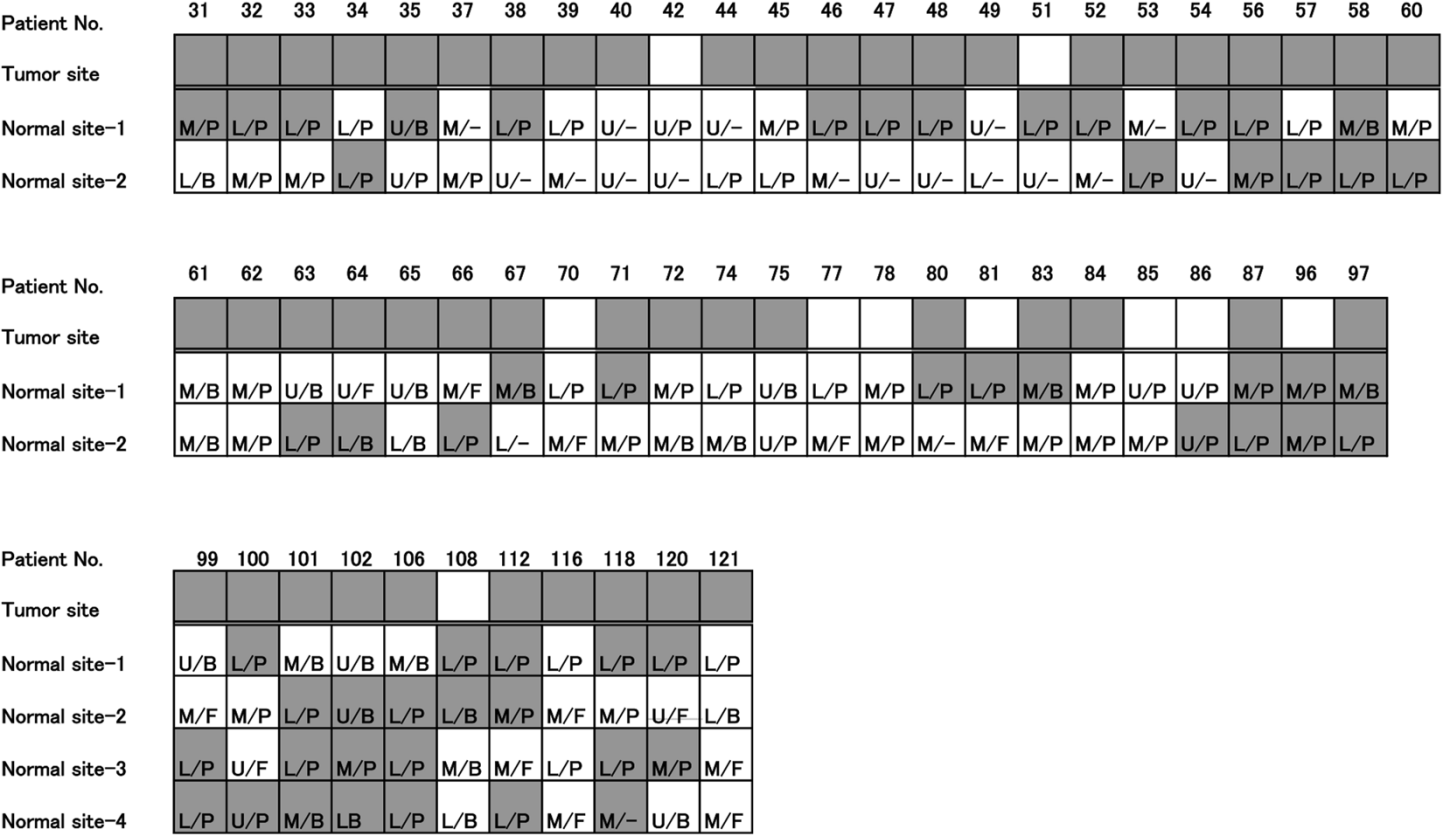
Expression of KK-LC-1 in normal areas evaluated in the corpus and gastric gland. Gastric specimens carrying a tumour were assessed for the expression of KK-LC-1 in two or four non-tumour areas. Each non-tumour area was distinguished as the upper (U), middle (M), or lower (L) corpus, and fundic (F), borderline (B), pyloric (P), or undistinguished (−) gastric glands.

By examining KK-LC-1 expression at one non-tumour site in gastric cancer specimens, KK-LC-1 was detected in five out of 24 (20.8%) specimens (Fig. 1a). At two non-tumour sites, KK-LC-1 was detected in 30 out of 48 (62.5%) specimens (Fig. 2a). At four non-tumour sites, KK-LC-1 was detected in nine out of 11 (81.8%) specimens (Fig. 2b).

### KK-LC-1 expression in gastric corpus/gland subsets

Non-tumour sites of gastric cancer specimens were divided into the upper (U), middle (M), and lower (L) gastric corpus, and/or fundic (F), borderline (B), and pyloric (P) areas of gastric glands (Fig. 2). The KK-LC-1 expression rate of L (66.1%) was significantly higher than that of M and U (27.5 and 14.3%, respectively, p < 0.01, Table 2). KK-LC-1 was not detected at F. Its expression rate in P (59.2%) was significantly higher than that in F (p < 0.01, Table 3). The subset of L/P had significantly higher KK-LC-1 expression (36 positives in 47 samples, 76.6%) than whole samples except for those in L/P subset (20 positives in 62 samples, 32.3%, p < 0.01, Table 4). The difference in these data were not considerable between two and four sampling points of a specimen.

**Table 2:**
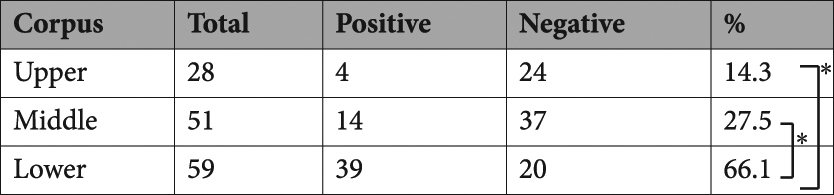
Expression of KK-LC-1 in gastric corpus. *p < 0.01 by Fisher’s exact test.

**Table 3:**
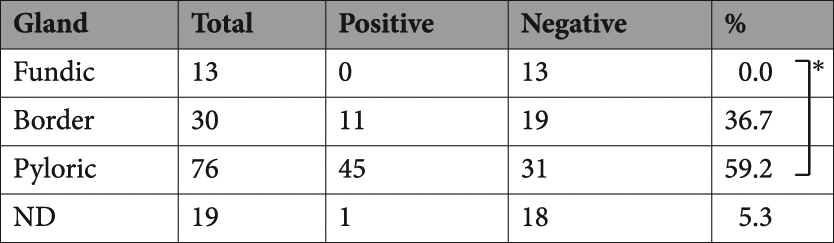
Expression of KK-LC-1 in gastric gland. ND: not distinguishable. *p < 0.01 by Fisher’s exact test.

**Table 4:** Expression of KK-LC-1 in gastric gland and corpus.

corpus	The expression of KK-LC-1											
	Fundic gland (F)			Border line (B)			Pyloric gland (P)			All		
	Positive	Negative	rate (%)	Positive	Negative	rate (%)	Positive	Negative	rate (%)	Positive	Negative	rate (%)
Upper (U)	0	3	0.0	2	6	25.0	2	5	28.6	4	14	22.2
Middle (M)	0	10	0.0	6	9	40.0	7	15	31.8	13	34	27.7
Lower (L)	0	0	—	3	4	42.9	36	11	76.6*	39	15	72.2
All	0	13	0.0	11	19	36.7	45	31	59.2	56	63	47.1

The significant difference of L/P subset was compared to whole specimens except those in L/P subset.

*p<0.01 by Fisher’s exact test.

### Correlation between KK-LC-1 expression and intestinal metaplasia

The 76 samples, which were in the pyloric gland, were assessed for KK-LC-1 expression and the occurrence of intestinal metaplasia (IM). There was a significant difference in KK-LC-1 expression between the IM-positive area (66.7%) and IM-negative area (32.0%, p < 0.01, Table 5).

**Table 5:**
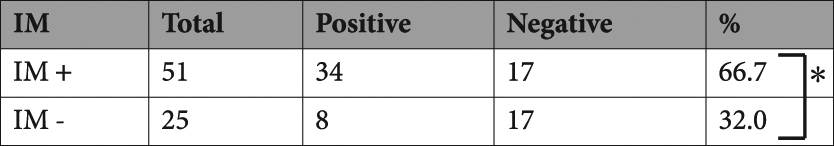
Expression of KK-LC-1 with intestinal metaplasia in pyloric gland. IM, Intestinal metaplasia. *p < 0.01 by Fisher’s exact test.

## Discussion

CTAs are expressed in about a quarter of all tumours in a wide range of organs, such as melanomas, and lung, oesophageal, gastric, colon and breast carcinomas, but not in normal tissues except for germline tissues. KK-LC-1 is a CTA by this definition, because it is not expressed in normal tissues, except for the testis, but is expressed in cancers of multiple organs. However, we observed its expression at non-tumour sites of stomachs carrying a gastric tumour. This study is the first report of its expression in non-tumour area of stomach. Paret *et al*. confirmed that KK-LC-1 is not expressed in the stomachs of healthy individuals^11^. Although the MAGE-A1 expression rate is 80% in HCC, which is the same as the KK-LC-1 expression rate in gastric cancer, it is not expressed in the non-tumour area of a liver carrying a tumour^15^. Although our study indicated that MAGE-A1 was expressed at a non-tumour site, it was only in one out of 98 samples, and thus was a rare case. There is the possibility that KK-LC-1 may be a CTA expressed during a precancerous stage.

A recent study has indicated that CTAs are expressed in oesophageal dysplasia, although CTAs are considered to be not or rarely expressed in non-tumour sites^16^. This result suggests that a specific CTA is expressed during precancerous stages in the specific tissue. The infection of *H. pylori* induces pathological abnormalities such as metaplasia, dysplasia, and adenocarcinoma^2,17^. Moreover, *H. pylori* induces expression of a CTA, and its infection correlates with KK-LC-1 expression in gastric cancer^12,13^. In particular, the rate of KK-LC-1 expression is high in not only advanced stage cancer, but also the early stage of gastric cancer^14^. In addition, KK-LC-1 is induced by *H. pylori* infection and maintains its expression during the occurrence and progression of gastric cancer.

It is known that accumulation of epigenetic alteration is one of the causes for occurrence of malignancy. Especially in stomach, the descending order of those accumulation, which is mainly induced by *H. pylori* infection, is gastric cancer tumour, non-tumour area of stomach carrying a tumour, atrophic gastritis with *H. pylori* infection and stomach without *H. pylori* infection^18^. The expression of CTAs is also occurred with accumulation of epigenetic alteration^19^. The expression of KK-LC-1 might start at meaningful amounts of epigenetic alteration but not enough to the amounts to achieve malignancy.

KK-LC-1 was frequently detected in the lower corpus including the antrum and pyloric gland. *H. pylori* colonise the pyloric antrum and extend to other areas of the stomach. *H. pylori* exist on gastric mucosa and in pyloric glands, but not in IM and tumour areas^20^. It has been reported that metachronous cancer developing from gastritis mucosa of the gastric remnant occurs in 30% of patients after endoscopic mucosal resection/endoscopic submucosal dissection^21^. In addition to detection of KK-LC-1 at the pyloric gland of gastric cancer specimens and correlation of KK-LC-1 expression with *H. pylori* infection, the hypothesis has arisen that *H. pylori* colonise and affect the pyloric gland for cancerisation. Between the infection and cancerisation, KK-LC-1 expression might be induced. KK-LC-1 expression before malignancy might be advantageous for detection and prediction of precancerous cells expressing KK-LC-1.

KK-LC-1 was significantly expressed in the observed IM area. *H. pylori* induce IM in the pyloric gland, but are distant from the IM area after occurrence of IM, indicating that KK-LC-1 was expressed after *H. pylori* infection and maintained expression after the absence of *H. pylori*. Conversely, our results also revealed that KK-LC-1 was expressed in non-IM areas, which was in the minority of cases. IM was a dominant event in stomachs carrying a gastric tumour. However, about 30% of patients with stomachs carrying tumours do not have IM^17^, which is similar to the rate of non-IM in KK-LC-1-expressing lesions. The similarity between IM and KK-LC-1 expression is that both were correlated to *H. pylori* infection, but they were not completely related to each other. Hattori *et al*. advocate the concept that *H. pylori* independently induce the metaplastic changes into either IM, dysplasia, or adenocarcinoma, and that some IMs and dysplasias lead to adenocarcinoma without *H. pylori*^17^. IM is not a precancerous lesion, but a paracancerous lesion^22^. Considering these facts, KK-LC-1 may be more closely related to the precancerous phenomena than IM.

CTAs including KK-LC-1 are considered as therapeutic targets rather than diagnostic targets for various kinds of cancer, because they are expressed in cells that reach a cancerous stage. Our study indicates that KK-LC-1 may be applicable as not only a therapeutic target but also a diagnostic and predictive marker for gastric cancer because of its frequent expression in non-tumour lesions of stomachs carrying a tumour.

## Methods

The study protocol was approved by the Human Ethics Review Committee of Kitasato University Medical Center, Japan (Approval No. 28-21) and all the experiments were carried out in accordance with relevant guidelines and regulations. Signed informed consent was obtained from all patients prior to collection of the tissue samples used in this study.

### Patients

A total of 177 patients underwent surgical resection for gastric cancer at the Department of Surgery, Kitasato University Medical Center, Kitamoto, Japan, between May 2011 and October 2015. Before the resection, we obtained a signed informed consent from each patient. We obtained 121 sets of a tumour samples and 1–4 non-tumour sample(s) from the gastric specimens after the surgical resection. Thirty-nine sets were omitted because tumour cells were not predominantly included in the sampled tumour area or did not achieve the standard of mRNA quality. Finally, 82 sets were used in this study. Eighty-two tumour and 138 non-tumour samples are assessed for expression of KK-LC-1. The clinicopathological findings were classified according to the Japanese Classification of Gastric Carcinoma (14^th^ edition)^23^. The background characteristics of the patients are shown in Supplementary Table 1.

### Tissue specimens

**Figure 3:**
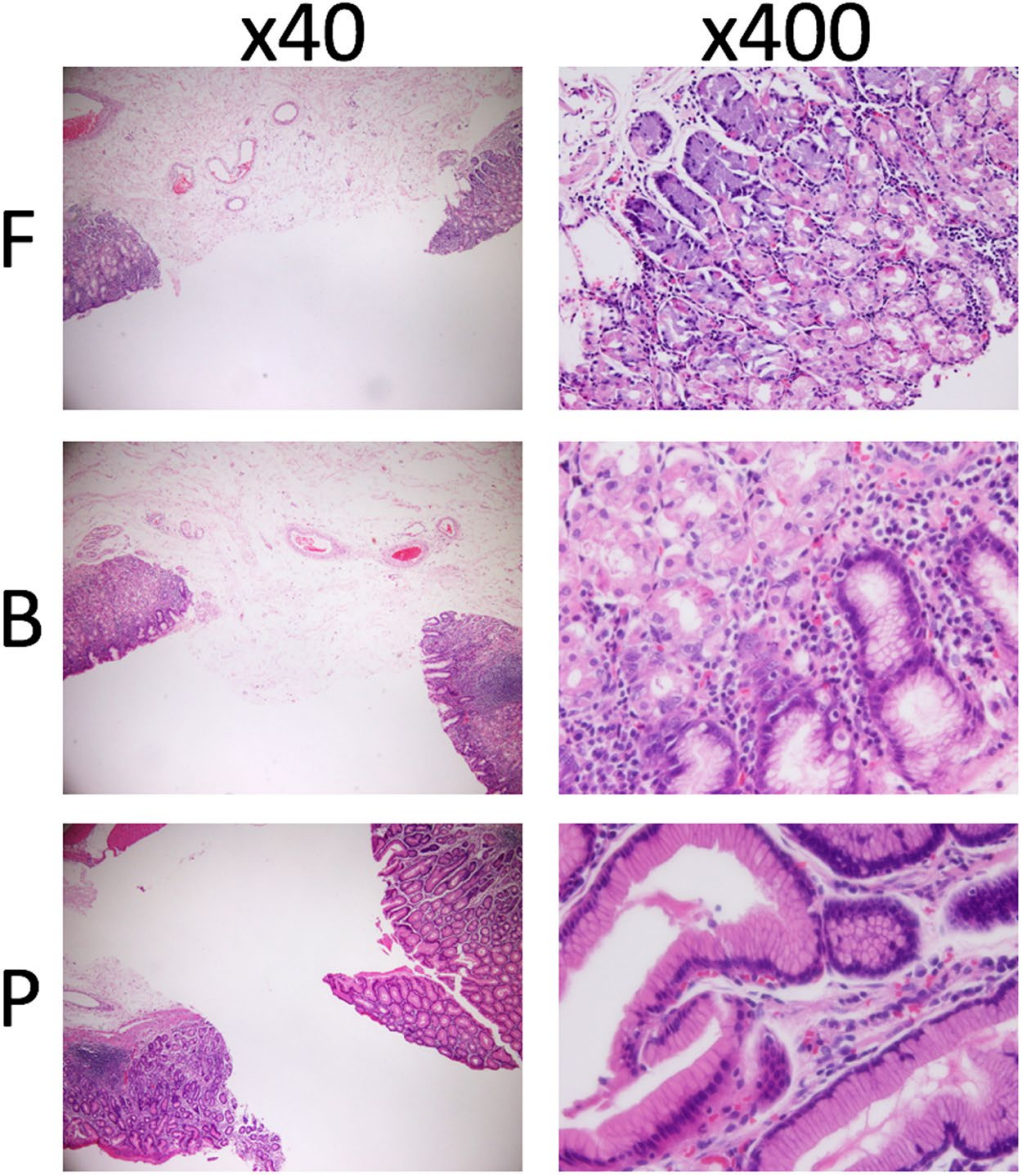
Haematoxylin and eosin staining to assess gastric glands. All non-tumour areas including the sampled area were subjected to haematoxylin and eosin staining. The adjacent sampled gland area was distinguished as the pyloric gland (P), fundic gland (F), or their combination (borderline; B). When both ends were all P or F glands, the sampled area was considered as P or F. When one end was observed as a combination of P and F glands, the sampled area was considered as B. If one end was P and the other was F, the sampled area was considered as B.

Tumour and non-tumour samples were immediately stored at 4 °C overnight in RNAlater (Life Technologies, Carlsbad, CA) and then stored at −80 °C until use. In macroscopic observation, each non-tumour sample was distinguished into the lower, middle, or upper corpus according to the Japanese Classification of Gastric Carcinoma (14^th^ edition)^23^. Each adjacent area sampled for assessment of gene expression was subjected to haematoxylin-eosin staining to confirm the predominance of tumour cells in the tumour area and no contamination of tumour cells in the non-tumour area, and to distinguish the sampled non-tumour area into the fundic gland, borderline, or pyloric gland (Fig. 3).

### Expression of CTAs

Total RNA from each sample was isolated using a QIACUBE and RNeasy Tissue Mini Kit (Qiagen, Hilden, Germany), according to the manufacturer’s instructions. Total RNA was converted to cDNA using oligo p(dN)_6_ random primers and Superscript III reverse transcriptase (Life Technologies). The expression of β-actin, MAGE-A1, -A3 and -A4, NY-ESO-1, and SSX-4 was measured with TaqMan Gene Expression Assays (IDs: Hs99999903_m1, Hs00607097_m1, H200366532_m1, Hs00365979_m1, Hs00265824_m1, and Hs02341532_m1, respectively). Analyses were performed using a 7900HT Fast Real-Time PCR System (Life Technologies). The threshold cycle number of cDNAs converted from RNAs was measured for β-actin. Then, <28 threshold cycles were passed, and the samples were assessed for the expression of CTAs. Real-time PCR was performed in a 20 μL reaction containing 5 μL cDNA template, 10 μL FastStart Universal Probe Master Mix (Roche, Manheim, Germany), and 1 μL of TaqMan Gene Expression Assay. We examined the expression of KK-LC-1 by 40 cycle-end point RT-PCR because an appropriate probe to detect KK-LC-1 mRNA was unavailable. PCR amplification was performed in 20 μL PCR reactions containing 2 μL cDNA template, rTaq (Takara, Tsu, Japan), dNTPs (Roche, Basel, Switzerland), and 500 nM each of gene-specific primers ATGAACTTCTATTTACTCCTAGCGAGC and TTAGGTGGATTTCCGGTGAGG (Sigma-Aldrich Japan, Shinagawa, Japan). The annealing temperature was 67 °C, and 40 cycles were used to yield the 342 bp product. PCR products were visualised by ethidium bromide staining and ultraviolet light exposure after electrophoresis on 1.5% agarose gels.

### Statistical analysis

Statistical analyses between KK-LC-1 expression and each factor were performed using Fisher’s exact test. P-values of less than 0.01 were considered as significant. JMP8.0 (SAS institute Japan, Minato-ku, Japan) was used for the analysis.

### Data availability

All data generated or analysed during this study are included in this published article (and its Supplementary Information files).

## Electronic supplementary material

Supplementary table 1

## References

[1] Stewart, B. W. & Wild, P. C. editors *World Cancer Report 2014. Lyon, France: International Agency for Research on Cancer* (2014). 39432694

[2] Uemura N (2001). Helicobacter pylori Infection and the Development of Gastric Cancer. New England Journal of Medicine.

[3] Miki K (2011). Gastric cancer screening by combined assay for serum anti-Helicobacter pylori IgG antibody and serum pepsinogen levels — “ABC method”. Proceedings of the Japan Academy, Series B.

[4] Mizuno S (2010). Prescreening of a high-risk group for gastric cancer by serologically determined Helicobacter pylori infection and atrophic gastritis. Dig Dis Sci.

[5] van der Bruggen P (1991). A gene encoding an antigen recognized by cytolytic T lymphocytes on a human melanoma. Science.

[6] Scanlan MJ, Simpson AJ, Old LJ (2004). The cancer/testis genes: review, standardization, and commentary. Cancer immunity.

[7] Stevanovic S (2017). Landscape of immunogenic tumor antigens in successful immunotherapy of virally induced epithelial cancer. Science.

[8] Shigematsu Y (2010). Clinical significance of cancer/testis antigens expression in patients with non-small cell lung cancer. Lung Cancer.

[9] Fukuyama T (2006). Identification of a new cancer/germline gene, KK-LC-1, encoding an antigen recognized by autologous CTL induced on human lung adenocarcinoma. Cancer Res.

[10] Shida A (2015). Frequent High Expression of Kita-Kyushu Lung Cancer Antigen-1 (KK-LC-1) in Gastric Cancer. Anticancer research.

[11] Paret C (2015). CXorf61 is a target for T cell based immunotherapy of triple-negative breast cancer. Oncotarget.

[12] Fukuyama T (2012). Helicobacter pylori, a carcinogen, induces the expression of melanoma antigen-encoding gene (Mage)-A3, a cancer/testis antigen. Tumour Biol.

[13] Fukuyama T (2017). Correlation Between Expression of the Cancer/Testis Antigen KK-LC-1 and Helicobacter pylori Infection in Gastric Cancer. In vivo (Athens, Greece).

[14] Futawatari N (2017). Early gastric cancer frequently has high expression of KK-LC-1, a cancer-testis antigen. World J Gastroenterol.

[15] Yamashita N (1996). High frequency of the MAGE-1 gene expression in hepatocellular carcinoma. Hepatology (Baltimore, Md.).

[16] Chen YT, Panarelli NC, Piotti KC, Yantiss RK (2014). Cancer-testis antigen expression in digestive tract carcinomas: frequent expression in esophageal squamous cell carcinoma and its precursor lesions. Cancer Immunol Res.

[17] Hattori T (1986). Development of adenocarcinomas in the stomach. Cancer.

[18] Leodolter A (2015). Somatic DNA Hypomethylation in H. pylori-Associated High-Risk Gastritis and Gastric Cancer: Enhanced Somatic Hypomethylation Associates with Advanced StageCancer. Clinical and translational gastroenterology.

[19] Honda T (2004). Demethylation of MAGE promoters during gastric cancer progression. Br J Cancer.

[20] Miyata H (1999). Distribution of Helicobacter pylori in a Mongolian gerbil gastric ulcer model. Laboratory animal science.

[21] Kobayashi M (2014). Clinicopathological Evaluation of Metachronous Gastric Cancers after ESD. Stomach and Intestine.

[22] Yao, T., Ueyama, H. & Hirahashi, M. High Risk Group of the Gastric Carcinogenesis from the Pathological Point of View: with Special Reference to Mucin Phenotypic Expression of the Carcinoma and Its Background Mucosa. *Stomach and Intestine*, 10.11477/mf.1403101740 (2009).

[23] Japanese classification of gastric carcinoma: 3rd English edition. *Gastric Cancer* **14**, 101–112, 10.1007/s10120-011-0041-5 (2011). 10.1007/s10120-011-0041-5 21573743

